# Hunting Poses Only a Low Risk for Alveolar Echinococcosis

**DOI:** 10.3389/fpubh.2019.00007

**Published:** 2019-01-29

**Authors:** Monika Wetscher, Klaus Hackländer, Viktoria Faber, Ninon Taylor, Herbert Auer, Georg G. Duscher

**Affiliations:** ^1^Department of Integrative Biology and Biodiversity Research, Institute of Wildlife Biology and Game Management, University of Natural Resources and Life Sciences, Vienna, Austria; ^2^Third Medical Department with Haematology, Medical Oncology, Haemostaseology, Infectious Diseases and Rheumathology, Oncologic Center, Paracelsus Medical University, Salzburg, Austria; ^3^Department of Medical Parasitology, Center of Pathophysiology, Infectiology and Immunology, Institute of Specific Prophylaxis and Tropical Medicine, Medical University Vienna, Vienna, Austria; ^4^Department of Pathobiology, Institute of Parasitology, University of Veterinary Medicine, Vienna, Austria

**Keywords:** *Echinococcus multilocularis*, active hunters, serological screening, risk factor, Austria

## Abstract

The Austrian province of Tyrol belongs to the areas where the alveolar echinococcosis (AE) caused by the fox tapeworm *Echinococcus multilocularis* (*E. multilocularis*) is highly endemic. In Central Europe and since 2011 in Austria, a growing incidence of human cases of AE has been observed, presumably linked with increasing fox populations infected by the fox tapeworm *E. multilocularis*. Hunting and the related activities put hunters in a high-risk group, and they are considered particularly vulnerable for the contraction of an AE. In light of this risk and the increased number of AE cases made public in Austria, the objective of the study was to investigate the prevalence of AE in hunters and to provide a possible connection to the incidence increase. In 2015 and 2016, we examined 813 serums of active hunters from all nine districts of Tyrol and serologically tested them for *E. multilocularis* antibodies. Twenty-one (2.58%) positive results in ELISA were detected via Western blot (WB), and only one (0.12%) serum showed a low positive reaction. No lesion in the liver parenchyma could be detected by abdominal ultrasonography in this patient so far, but the risk of developing alveolar echinococcosis remains for this WB-positive hunter. Risk factor analysis of these 813 hunters revealed that 697 (85.7%) hunted red foxes regularly and 332 (40.8%) of those skinned them as well. Three hundred and eighteen (39.1%) out of the 813 hunters were owners of hunting dogs; 89 (10.9%) and 243 (29.9%) were owners of non-hunting dogs and cats, respectively. Our results indicate that hunters do not have a greater risk of infection with *E. multilocularis* compared to non-hunters in Austria. The cause of the unexpected increase in AE cases in Austria remains unclear.

## Introduction

Human alveolar echinococcosis (AE) is caused by the metacestode stage of the fox tapeworm *Echinococcus multilocularis* (1). In Central Europe, this rare parasitic disease that is potentially fatal in humans (2), primarily circulates within a sylvatic life cycle comprising red foxes (*Vulpes vulpes*) as definitive hosts and rodents (e.g., *Microtus arvalis, Arvicola terrestris, Ondatra zibethica*) as intermediate hosts (3, 4). The raccoon dog (*Nyctereutes procyonoides*) as neozoon species plays a similar role as the red fox as final host (4, 5). Also, pets, especially dogs, can also act as final hosts and contaminate the environment by excreting infective *E. multilocularis* eggs (6–10). Humans acquire the infection by peroral ingestion of *E. multilocularis* eggs present in contaminated soil, food, or animal skins (6, 11, 12). Humans acquire the infection by peroral ingestion of *E. multilocularis* eggs present in contaminated soil, food, or animal skins (6, 11, 12). The *E. multilocularis* metacestode in the intermediate host establishes itself primarily in the liver and shows a tumor-like growth pattern similar to a malignant tumor (13–15). Due to the prolonged growth of the parasite, the lack of specific symptoms in many cases, and the long incubation period of up to 20 years, AE is often diagnosed and treated in a rather late and metastatic stage (11, 16). Serological tests for detection of AE are available and allow early detection of disease before clinical manifestation (1, 17).

During recent decades the fox population increased due to successful rabies vaccination (18, 19). Hunting has repeatedly been published as a risk factor (20–24) and associated with AE acquisition (25). Further, there has been an increasing incidence of human AE cases in Austria, especially in the western provinces of Austria (Tyrol and Vorarlberg) (23). These increased AE cases were unexpected. Thus, it was recommended to improve the surveillance system in Austria by screening exposed individuals such as hunters, especially in *E. multilocularis*-endemic regions, to detect early AE cases (23).

To obtain data on the current situation regarding the aforementioned epidemiological development, we performed a serological study on anti-*E. multilocularis* antibodies comprising hunters of all nine Tyrolean districts in 2015 and 2016. This particular group was selected because of their potentially high exposure to eggs from infested foxes through their hunting activity, with the aim of quantifying human AE prevalence in hunters.

## Materials and Methods

### Study Area

The study area is the province of Tyrol with its nine districts. Tyrol is bordered by the provinces of Salzburg to the east, Vorarlberg to the west, and Carinthia to the south. The neighboring countries of Tyrol are Germany (to the north), Italy (to the south), and Switzerland (to the west).

### Sample Collection

The Hunting Association of Tyrol invited their members to participate at the annual meetings for the display of trophies in the capital cities of their districts. Out of 16,146 active hunters, 813 (i.e., 5%, 736 males and 77 females) were willing in 2015 and 2016 to provide blood samples at these events.

Approval for this study was obtained from the Ethics Committee in Salzburg on January 28th, 2015 (No: 415-E/1845/2-2015). The written informed consent was given by the patients for their information to be stored and used for this study.

For demographic data and risk factors, each participant was also asked to complete a questionnaire concerning their hunting activity. The issues raised were temporal hunting activity, killing of red foxes, skinning of foxes as well as hunting dog ownership. Further questions concerned the ownership of pets such as dogs or cats.

The venous blood samples taken by medical doctors were performed using Vacuette®–Multiple Use Drawing Needle (Greiner Bio-One GmbH. 4,550 Kremsmünster, Austria) and Vacuette®–8 ml Z Serum Sep Clot Activator (Greiner Bio-One GmbH. 4,550 Kremsmünster, Austria). The samples were stored at +5°C.

Serum tubes and questionnaires were numbered and checked twice by independent persons (medical doctors and a principal investigator) for their reliability.

### Serological Testing

The analysis of the sera for anti-*E. multilocularis* antibodies was performed within 48 h at the Specific Prophylaxis and Tropical Medicine (SPTM). As a basic test, an enzyme-linked immunosorbent assay (ELISA) with *E. multilocularis* crude antigen was used (26). A commercial western blot (WB, LDBio, France) was used to confirm the positive results (27). Serum samples were considered serologically positive if they showed clearly positive reactions in the ELISA (cut-off: 20 Antibody Units based on a positive control serum with 100 AUs). Persons with positive antibody values were examined by WB and were informed by the principal investigator. In addition, they were asked to provide a further blood sample, and in the case of verification of positive test results, the participants were again personally contacted and ultrasound/computed tomography of the liver was recommended.

### Data Analysis

All available details about age, hunting activities, pet ownership, and serological test were logged into an Excel spreadsheet (Microsoft Office Excel, Redmond, USA) and analyzed.

## Results

In total, 813 (736 males and 77 females) sera were evaluated during the 2 years human survey. The mean age of the hunters was 52.5 years and ranged from 18 to 86. The age distribution was as follows: 4.7% were 18–25 years old, 23.1% were 26–45 years old, 44% were 46–60 years old, and 28.2% were older than 60 years of age. All of the participants had been hunting for, at least, 1 year, 26.6% for 2–10 years and 20.8% for 11–20 years, whereas 52.6% had been actively hunting for more than 20 years.

Out of 813 hunters, 697 (85.7%) were regularly hunting red foxes, and 332 (40.8%) of these also skinned foxes. About 318 (39.1%) of the hunters had currently or in the recent past a hunting dog, and 89 (10.9%) hunters owned a non-hunting dog. Cat ownership was reported among 243 (29.9%) of the hunters (Table 1).

**Table 1:** Number of examined hunters in the nine Tyrolean districts, their hunting activities, and pet ownership.

**District**	***n* (%)**	***n* (%)**	***n* (%)**	***n* (%)**	**Active hunting period^**a**^**	***n*** **(%) ownership of pet**		
	**Examined hunters**	**Shoot foxes**	**Skinning foxes**	**Hunting dog owner**		**dog**	**cat**	**or both**
Innsbruck	37 (4.6)	28 (4.0)	13 (3.9)	12 (3.8)	17	6 (6.7)	8 (3.3)	3 (2.5)
Innsbruck-Land	98 (12.1)	84 (12.1)	30 (9.0)	32 (10.1)	21	8 (9.0)	27 (11.1)	11 (9.2)
Imst	43 (5.3)	41 (5.9)	20 (6.0)	20 (6.3)	29	2 (2.2)	15 (6.2)	10 (8.4)
Kitzbühel	98 (12.1)	92 (13.2)	49 (14.8)	32 (10.1)	26	16 (18.0)	31 (12.8)	13 (10.9)
Kufstein	123 (15.1)	111 (15.9)	56 (16.9)	47 (14.8)	25	11 (12.4)	38 (15.6)	18 (15.1)
Landeck	110 (13.5)	88 (12.6)	36 (10.8)	45 (14.2)	22	3 (3.4)	21 (8.6)	8 (6.7)
Lienz	119 (14.6)	98 (14.1)	38 (11.4)	52 (16.4)	22	18 (20.2)	42 (17.3)	26 (21.8)
Reutte	114 (14.0)	93 (13.3)	47 (14.2)	44 (13.8)	22	21 (23.6)	40 (16.5)	22 (18.5)
Schwaz	71 (8.7)	62 (8.9)	43 (13.0)	34 (10.7)	25	4 (4.5)	21 (8.6)	8 (6.7)
Total	813 (100.0)	697 (85.7)	332 (40.8)	318 (39.1)	23	89 (10.9)	243 (29.9)	119 (14.6)

a *Average of the active hunting period in years*.

The serological analysis for antibodies against *E. multilocularis* antigen showed a low positive ELISA result in 21 (2.6% participants) (Table 2 shows the hunting activities of the positive ELISA hunters), and one serum was positive by both ELISA and WB (Table 3). This serum originated from a male hunter from the district of Lienz (seroprevalence: 0.12%), and the abdominal sonography showed no lesion changes in the liver parenchyma. This 80 years-old hunter was hunting for 62 years, shot and skinned foxes regularly, and had neither a hunting dog nor pets regularly. The real prevalence among the hunters in this cohort is 0 due to the lack of any confirmed AE case.

**Table 2:**
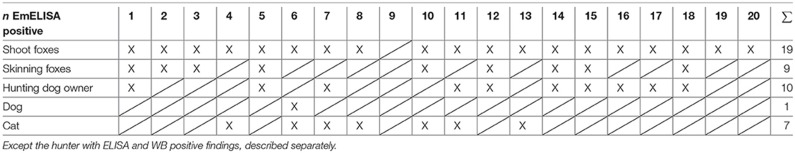
List of the 20 EmELISA positive hunters and their hunting activities and pet ownership.

*Except the hunter with ELISA and WB positive findings, described separately*.

**Table 3:** Number of licensed and examined hunters in the nine Tyrolean districts and their serological test results.

**District**	**^**a**^n of hunters**	***n* (%) of hunters examined**	***n* (%) of hunters ELISA positive**	***n* (%) of hunters WB positive**
Innsbruck	599	37 (4.6)	2	0
Innsbruck-Land	2.985	98 (12.1)	1	0
Imst	1.880	43 (5.3)	3	0
Kitzbühel	1.499	98 (12.1)	2	0
Kufstein	1.582	123 (15.1)	7	0
Landeck	1.988	110 (13.5)	1	0
Lienz	2.006	119 (14.6)	4	1
Reutte	1.262	114 (14.0)	0	0
Schwaz	2.345	71 (8.7)	1	0
Total	16.146	813 (100.0)	21 (2.58)	1 (0.12)

a *Licensed hunters per district*.

One of the 813 Tyrolean hunters (district Kitzbühel) had previously been treated successfully by surgical intervention (in-toto-extirpation of the parasite) in 2011. In our screening study, however, he presented a negative test result.

## Discussion

In the years 2015 and 2016, we tested the sera of 813 hunters from the province of Tyrol to evaluate the current situation of AE in the presumed risk group “hunters” originating from a highly endemic area (1, 7, 13). However, AE is still a life-threatening disease for humans (11), but the prognosis is improved if the infection is diagnosed at a very early stage (even long before clinical manifestation). The serum sample of an 80 years-old hunter from the district of Lienz yielded a positive result by ELISA and by WB. In this case an infection with AE can be assumed and further confirmed by using ultrasound as the imaging screening method of choice (28). So far no visible lesions in the liver of this patient could be found. Thus, the hunter was instructed to repeat serological tests as well as ultrasound examination twice a year during the following years. In effect, liver lesions can take years to become apparent in seropositive persons (29), and lesions <2 cm are difficult to diagnose (30). Due to its higher accuracy in detecting liver lesions [although without complete proof (31)], computerized tomography could be applied and was suggested but not carried out in this case, as this is the responsibility of the general practitioner.

Also, among another 20 hunters from all other districts (except in Reutte), low positive antibody levels could be determined, which could not be confirmed by WB. In these cases, it is necessary to carry out follow-up investigations to exclude an early stage of infection (1, 32), at least, once a year during the next 2 years (17, 29). Another explanation for this finding could be a cross-reaction in the ELISA (33). We can exclude *E. granulosus* in all 20 ELISA and WB positive *E. multilocularis* results by testing with indirect haemagglutination test (IHA) (26).

Although 792 hunters (97.4%) were serologically negative, documenting that they were not infected currently, all were strongly encouraged to re-examine their *Echinococcus*-immune status individually in intervals of 2–3 years, at least, during their active hunting period. The observation of seroconversion from negative to positive would demonstrate that a fresh infection must have occurred since the last examination.

If particularly exposed persons are regularly monitored by ultrasound imaging or by serological screenings (1, 34), the development of the slow-growing metacestode is rather limited and a cure becomes more likely. Hunters were considered as one of these risk groups (20–25) based on regular contact with foxes and pets (6, 35) as well as with contaminated fur (12) and environment.

In this study, 86% of hunters reported shooting foxes and 41% admitted to skinning foxes regularly. Also, 39% keep hunting dogs and up to 30% are owners of dogs or cats as pets. Despite these high-hazard exposure potentials due to long hunting activities, as almost 52.6% had been hunting for more than 20 years, there was no clear evidence to make a diagnosis of AE. Similar to a study of trappers with high hunting activity in South Dakota, none of the trappers showed antibody evidence for the presence of *E. multilocularis* (36). In a case-control study from Tyrol, hunting was published as an independent risk factor in relation to AE (25). In contrast, (7) were not able to prove a connection between hunting and AE with double the number of test subjects in neighboring Germany. Different methods give differentiated statements about the risk of hunting; case-control studies increase the probability that hunting is a risk factor, but not significantly. This was shown in a recently published study in which case-control and cross-sectional studies were compared and no connection between hunting and AE Echinococcosis was proven (37). In south Gansu, China, a large number of AE cases was diagnosed using serological screening and ultrasonography, and hunting had no effect as a risk factor (38). A total of 23,321 people were examined in three published serological screening studies in Austria (21, 39, 40). Two AE cases could be diagnosed with this preventive screening examination; both cases occurred in the province of Tyrol and had no connection to hunting. Given these results, we assume that hunters are not at a higher risk of contracting an AE than non-hunters. Furthermore, the increase in confirmed AE cases in Austria could not be ascribed to hunting due to a lack of available data on occupation and leisure activities (23). The study of 149 (100%) hunters in south-eastern Austria showed 7 (5%) subjects with a positive ELISA value and none could be confirmed in WB (21). In our studies, with a 5.5 times higher number of test subjects, there were 2.6 and 0.12%, ELISA and WB, respectively, positive results. The ELISA reactions show that increased contact with *E. multilocularis* eggs (41) takes place due to hunting. In healthy individuals, however, only a low level of manifestation takes place and an AE is prevented (42). In contrast, a weakened immune system promotes infection of *E. multilocularis* in humans (43, 44) and significantly increases the risk of contracting AE (45, 46). In the course of this study, we tested one individual whose AE liver lesion was surgically removed in 2011. The lack of antibodies in this subject is presumably due to the success of the surgery (47). Furthermore, it should be mentioned that this test subject suffered from cancer and had immunosuppressive therapy beforehand.

Studies show that pets, dogs, and cats excrete infectious *E. multilocularis* eggs (6, 8–10). The case-control study from Tyrol, in which hunting poses a risk, also showed a connection between cats and AE in western Austria (25). In this single case-control study in Austria, dog ownership was reported as a low-risk factor. In contrast, another case-control study showed dog ownership, especially where the dogs spend a lot of time outdoors, as a major risk factor (7). An increase in human seroprevalence was reported in an area with a high prevalence of *E. multilocularis* in intermediate hosts (rodents) and prevalence detection of *E. multilocularis* in domestic animals, however, without an increase in human AE cases (48). In our study, 69% of test subjects had a hunting dog as well as dogs and cats as pets. The potential risk of infection is unknown in Austria, as there is no data on the prevalence of *E. multilocularis* in dogs and cats. Our study shows that despite considerable possession of hunting dogs and pets, no AE was diagnosed in the risk group of hunters.

To summarize, the results indicate that hunters come into contact with *E. multilocularis*, which was ascertained by positive ELISA and WB tests. However, thus far, we have not been able to observe that the proportion of hunters is higher than the proportion of non-hunters among all Austrian AE patients. The unexpected AE cases in Austria could not be explained with our investigations of an exposed group of “hunters.” This study demonstrates the need for further research to elucidate on conflicting results and identify the actual risk factors for AE to be able to avoid preventable AE illnesses. Missing data, especially in pets about the prevalence rates of *E. multilocularis* in Austria, should be investigated in future.

## Ethics Statement

This study was carried out in accordance with the recommendations of the Ethics Committee in Salzburg. All subjects gave written informed consent in accordance with the Declaration of Helsinki. The protocol was approved by the Ethics Committee in Salzburg on January 28th, 2015. 415-E/1845/2-2015.

## Author Contributions

MW designed the study. MW and VF were responsible for taking blood samples. MW, KH, HA, VF, NT, and GD all contributed to the manuscript editing and approved the final version of this manuscript.

### Conflict of Interest Statement

The authors declare that the research was conducted in the absence of any commercial or financial relationships that could be construed as a potential conflict of interest.
